# Reducing inﬂuenza spreading over the airline network

**DOI:** 10.1371/currents.RRN1005

**Published:** 2009-08-21

**Authors:** Jose Marcelino, Marcus Kaiser

**Affiliations:** ^*^School of Computing Science - Newcastle University and ^†^Newcastle University

## Abstract

Disease spreading through human travel networks has been a topic of great interest in recent years, such as with swine inﬂuenza or SARS pandemics.

Most studies have proposed removing highly connected nodes (hubs) to control spreading. Here, we test alternative strategies using edge removal (ﬂight cancellation) for spreading over the airline network. Flight cancellation was more efﬁcient than shutting down whole airports: spreading took 81% longer if solely selected ﬂights were removed, compared to a 52% reduction when entire airports were shutdown, affecting the same number of ﬂights.

## Introduction

Complex networks are pervasive and underlie almost all aspects of life. They appear at different scales and paradigms, from metabolic networks, the structural correlates of brain function, the threads of our social fabric and to the larger scale making cultures and business come together through global travel and communication. Recently, these systems have been modeled and studied using network science tools giving us new insight in ﬁelds such as sociology, epidemics, systems biology and neuroscience. Typically in such systems major components such as cities are modeled as nodes and functional or structural connections - flights, for example - between such components are represented as edges. Many such networks were shown to be small-world [1] with higher neighborhood connectivity compared to Erdős–Rényi random networks [2]. Some networks are scale-free containing highly connected nodes (hubs) and having a power-law degree distribution. In these networks, the probability of a node having \begin{equation*}k\end{equation*} edges follows a power law \begin{equation*}k^{-\gamma }\end{equation*} [3]. It is possible for a network to show both scale-free and small-world properties, however the two features may also appear independently. In addition, small-world networks may or may not contain multiple clusters or communities. The relation between changes in network topology and the resulting structural integrity, as measured by characteristic path length or global efﬁciency [4], gives an indication of the robustness towards failure in connected systems. Many studies have looked into the error and attack tolerance of these networks regarding the removal of nodes [5] [6]. For scale-free networks, the selective inactivation of hubs had a much greater impact on structural network integrity than simply removing randomly selected nodes [6]. Spreading on such heterogeneous networks could be impeded by targeting hubs as well [7]. Structural network integrity could also be inﬂuenced by partially inactivating speciﬁc connections (edges) between nodes [8] [9] [10]. In this article we consider the dynamic effect of topological changes as measured by the time it takes until an epidemic spreading process reaches half of a network. Spreading starts from one infected node and progresses through connections to susceptible nodes as in the standard Susceptible-Infected (SI) model [11]. By using this model, combined with different strategies for predicting critical edges, we determined how the removal of edges slows down the spreading dynamics.Comparing a range of removal strategies against the established hub removal we ﬁnd that removing selected edges has a bigger impact on network spreading activity with signiﬁcantly lower number of removed connections. For the global airline network this shows that only a smaller set of ﬂights would need to be stopped instead of canceling all the ﬂights from a set of airports (see Fig. 1 with Mexico City as starting node of an outbreak). In addition we also found that community structure plays a critical role in spreading and not the degree distribution and this method of slowing spreading by removing critical, higher ranked, connections is particularly effective in ﬁnding the links that connect such communities. Finally, we discuss the computational complexity of all strategies. Whereas some strategies are computationally costly for large or rapidly evolving networks, several edge removal strategies are as fast as hub removal while offering much better spreading control.

**Figure:**
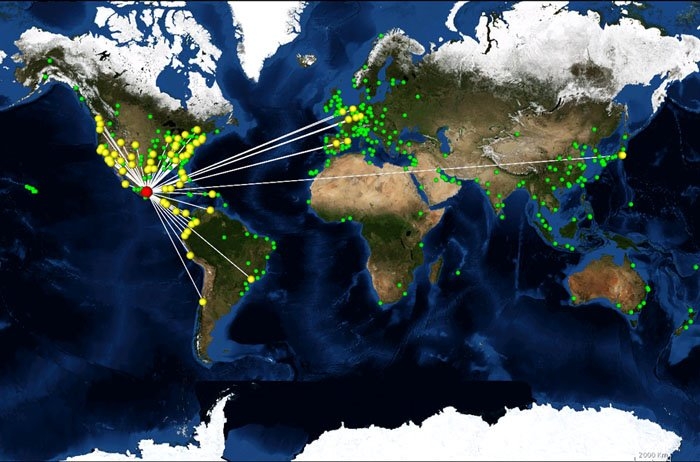
Figure 1. Spreading over the airline network with Mexico City as starting node (red). Nodes in yellow are directly connected whereas nodes in green are airports not directly linked to the starting point.

****

## 1. Results

Network spreading simulations starting at any node were summarized by T_1/2_, the average number of time steps for infecting half the nodes of a network. Spreading control strategies were evaluated by removing up to 25% of the edges and measuring the resulting relative increase of T_1/2_ (see Methods and Fig. 3).For the airline network used in the study, airports formed the nodes and an edge connects two airports if a scheduled ﬂight between them existed. Spreading in this network could show how a disease outbreak, e.g. SARS, would spread around the world [5].

Due to our methodology any airport can be an initial node for the outbreak, in contrast to other studies where only one airport could be the starting point [5] [12]. While this approach increased the variance in the resulting spreading times, it also offered additional insight into the effectiveness of removal strategies over a much wider range of outbreak scenarios.

**Figure:**
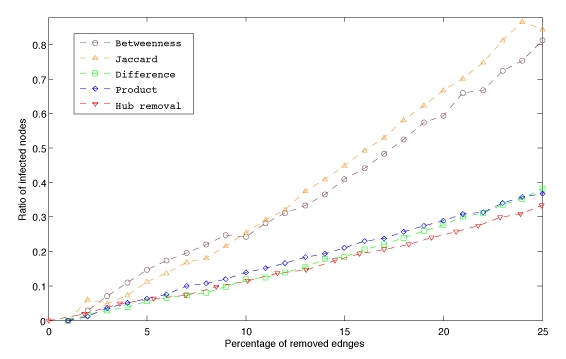
**Figure 2**. Spreading time versus sequential edge elimination by betweenness centrality, Jaccard coefficient, difference and product of degrees and by hub removal (see Methods). For example, a 0.8 ratio indicates that spreading took 80\% longer  than the intact case after the corresponding percentage of edges were removed.

Measures based on edge betweenness and Jaccard coefﬁcient were the two best predictors of critical edges (Fig. 2). Among the top intercontinental connections identiﬁed by betweenness are ﬂights from São Paulo (Brazil) to Beijing (China), Sapporo (Japan) to New York (USA) and Montevideo (Uruguay) to Paris (France). After removing a quarter of all edges, both strategies showed an increase of 82.5% and 88% in spreading time respectively, compared to only 33.3% for the hub removal strategy. Whereas in [5] a control strategy based on removal of the top 2% of the cities was investigated, we have considered the effect of disabling the top 2% of airports ranked by number of connections. In our case this strategy would remove 17.3% of all ﬂights in the network, causing a 20.5% increase in spreading time. Comparatively, removing the same number of ﬂights selected by edge betweenness would produce a 48.2% slowdown.

To understand the underlying mechanism of these results we produced four different, rewired, versions of the original network, preserving the degree distribution, preserving the community structure, preserving both or preserving none (equivalent to a Erdős–Rényi random network) of these features.

Applying the same spreading simulations on these rewired versions of the network showed that the highest increase in spreading times were obtained on networks that preserved the original community structure and again when removing highest ranking edges (see Fig. 3). The increase was particularly higher (262% slower than the intact network) when the original community structure was maintained, but the links inside each community were randomized, losing the original degree distribution at the local level for nodes inside each community.

**Figure:**
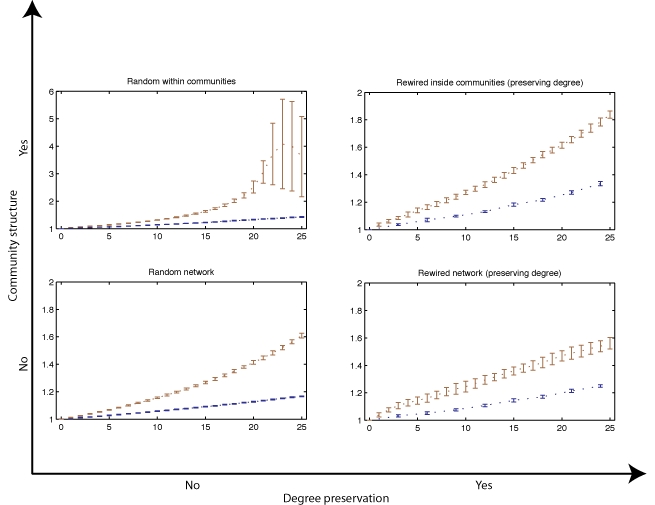
**Figure 3. **Spreading time over percentage of edges removed by edge betweenness centrality for four different rewired versions of the original airline network: fully randomized, randomized only within communities, preserving only the original degree distribution and preserving the degree distribution plus original communities. A spreading time ratio of 2 would mean spreading took twice the number of time steps compared to the intact network. Due to the greater spreading times observed for networks where links were randomized within communities (top left) a larger scale y-axis scale was used. 

The second highest increase was again observed for rewired networks that maintained the original community structure and degree distribution, where removing the same 25% of edges slowed down spreading by 83%, which is very similar to the results observed for the original airline network. Removing the same amount of edges from both networks that only maintained the original degree distribution, or indeed the fully randomized networks which did not maintain the communities or degree distribution showed similar slowdowns that fell within the standard deviation, at 56% and 60% respectively. Finally the strategy of removing hubs again showed to be very poor at slowing down spreading in these rewired networks, appearing consistently below the edge removal strategy. The highest slowdown for this strategy was obtained again in the network randomized inside communities with a 42% increase in spreading time. The smallest increase was observed in the fully random network, where the slowdown was only 17%. When preserving degree distribution the networks with the original communities slowed down on average 34% and without the communities 25%. For comparing single edge and hub removal strategies in these benchmark rewired networks, we considered the ratio between the slopes of the spreading time curves obtained as a result of the simulations. In all cases edge removal was at least twice as effective compared to removal of hubs with the same number of removed links. In randomized networks preserving the community structure the slope for edge removal was 5.6 times the one for hub removal. On the fully random network the edge removal slope was 3.70 times larger than hubs. For versions preserving the degree distribution, in networks containing the original communities the average edge removal slope was 2.31 time higher than hub removal and ﬁnally where only the degree distribution was preserved the edge removal to hub removal slope ratio was 2.17.

## 2. Discussion 

Selecting speciﬁc edges for removal can efﬁciently control spreading in the airline network with fewer “side effects” for the overall network structure compared to the traditional approach of removing highly connected nodes. With the same number of removed connections, edge removal strategies resulted in a larger slowdown of spreading than hub removal strategies. Edge betweenness was clearly superior at predicting the most critical edges; however, some of the less ideal measures were much faster to calculate. For large networks or networks where the topology frequently changes, such alternative fast measures will be useful. Edge betweenness was the computationally most intensive measure with \begin{equation*}O(n^{2}+e)\end{equation*} for a network with \begin{equation*}n\end{equation*} nodes and \begin{equation*}e\end{equation*} edges. Alternative strategies – which still perform better than hub removal – are the Jaccard index with \begin{equation*}O(n^{2})\end{equation*} requiring approximately 65% of the computing time and both degree measures (product and difference) with \begin{equation*}O(n)\end{equation*} with around 40% of the computing time of edge betweenness, assuming an implementation of the graph as an edge list. Note that the latter two measures are comparable to the computation time for the hub removal strategy, \begin{equation*}O(n)\end{equation*}.Whereas node removal according to degree was the worst strategy in this study, node centrality might lead to better results. Indeed, previous ﬁndings [13] show that the most highly connected cities in the airline system do not necessarily have the highest node centrality. However, node centrality would be computationally as costly as edge betweenness. Connections identiﬁed by edge removal strategies were critical for the transmission of infections or activity and can be targeted individually with fewer disruptions for the overall network. In the transportation network studied, this would mean higher ranked individual ﬂights could be canceled instead of shutting down an whole airport. Results obtained from simulating the same spreading strategy over differently rewired versions of the airline network showed that this method of controlling spreading is particularly effective in slowing down spreading in networks that have a modular structure, as is the case for spatially distributed real-world networks [14] [15] [16]. These benchmark studies indicate that the effectiveness of edge removal in the airline network is due to its community structure rather than its power-law degree distribution.

## 3. Materials and methods 

All data sets are available on our website http://www.biological-networks.org.

### 3.1 Airline connections network 

As in previous studies [5] [13], we obtained scheduled ﬂight data for one year provided by OAG. This listed 1,341,615 records of worldwide ﬂights operating from July 1, 2007 to July 30, 2008, which is estimated by OAG to cover 99% of the commercial ﬂights. The records include the cities of origin and destination, days of operation and the type of aircraft in service for that route. Airports were uniquely identiﬁed by their IATA code and became the nodes in the network. Short-distance links corresponding to rail, boat, bus or limousine connections were removed from our data set. An edge connecting a pair of nodes is present if at least one scheduled ﬂight connected both airports. As in previous studies [5], we used a subgraph containing the 500 top airports which was obtained by selecting the airports with greater seat trafﬁc on all incoming and outgoing routes. This subset of airports still represents at least 95% of the global trafﬁc.

### 3.2 The spreading mode

**Figure:**
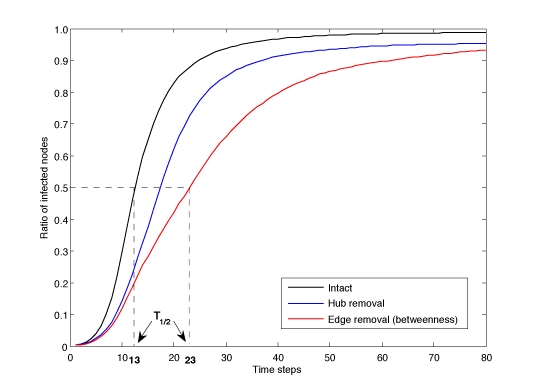
**Figure 4**. Spreading for Mexico City as starting node, measured by the ratio of infected nodes on  the intact network (black) and after removing 25% of edges by hub removal (blue) or edge betweenness (red). T_1/2_ represents the time step where half of the nodes are infected.

Due to the range of networks considered, we based our analysis on the Susceptible-Infected (”SI”) epidemic spreading model [11] simulating the spreading of activity through networks. Nodes can either be susceptible to infection (”S”) or be infected (”I”), with no recovered or removed state. Infection can spread through any of the edges of an infected node to its susceptible neighbors with a ﬁxed probability λ, which in our simulations was ﬁxed at \begin{equation*}\lambda\end{equation*} \begin{equation*}= 0.01\end{equation*} for all networks. This model tests the effectiveness of our strategies for cases where nodes do not recover, such as early stage epidemic outbreaks. As we focus on initial spreading time and not in its long-term evolution our results should be useful in the ﬁeld of disease epidemics [5] [17] [18]. A more detailed model has been used for simulating spreading over the airline network [5], including stochastic local dynamics in cities and introducing a recovered state (SIR model).However, for the purpose of determining candidate measures for edge importance in spreading, such a detailed model was not necessary. The previous study was focused on a speciﬁc outbreak of SARS (Severe Acute Respiratory Syndrome), so there only Hong Kong considered as its starting point. In our model, however, we systematically tested all possible nodes as starting points leading to higher computational costs and a need for a simpler spreading model.

### 3.3 Edge removal strategies 

Five candidate measures for predicting critical edges in networks were tested. The measures are based on range of different parameters including node similarity, degree and all pairs shortest paths. Edge betweenness centrality [19] [20] represents how many times that particular edge is part of the all-pairs shortest paths in the network. Edge betweenness can show the impact of a particular edge on the overall characteristic path length of the network; a high value reveals an edge that will increase the average number of steps needed for spreading. The Jaccard similarity coefﬁcient (or matching index) [21] [22] shows how similar the incoming and outgoing connections of two connected nodes are. A low coefﬁcient reveals a connection between two different network structures that might represent a ”shortcut” between remote regions. The absolute difference of degrees for the adjacent nodes is another measure of similarity of two nodes. A large value here indicates a connection between a network hub a more sparsely connected region of the network. The product of the degrees of the nodes connected by the edge is high when both nodes are highly connected (hubs). For testing the absolute difference and product of degrees we also considered the opposite removal strategy (starting with lowest values) but the results showed to be consistently under-performing when compared to all other measures (not shown). Finally, highly connected nodes will be detected and the nodes, and therefore all the edges or that node, will be removed from the network. Note that this is referred to as ’hub removal strategy’ whereas the impact is shown in relation to the number of edges which are removed after each node removal.

### 3.4 The simulation algorithm 

All simulations were implemented in Java (Sun Microsystems, USA) using the JUNG (http://jung.sf.net) graph framework for the graph data model and the measures were implemented in custom Java code. Results were further processed in MATLAB (R2008b, MathWorks, Inc. Natick, USA). Simulations were run in parallel on a 16-core HP ProLiant server. Edge betweenness centrality was implemented using the algorithm by Brandes [20]. Links were considered to be directed for all networks. Due to the number of all possible combinations we used a Monte Carlo approach and averaged results from 50 spreading runs for each starting node. All nodes of each network were tested as starting nodes. These spreading runs were repeated after the removal of edges following all ﬁve strategies. For example, for the airline network we considered the average of 25,000 results (50 runs for 500 starting nodes) for each edge removal metric at each percentage point.As 26 percentage points were observed (including 0%), in total 25,000×26 = 650,000 results were considered per measure. Starting from the intact network, with the edges ordered by the selected measure, the simulation algorithm is as follows:

1.  From the graph \begin{equation*}G=\{V,E\}\end{equation*}, a single node \begin{equation*}v\in V\end{equation*} is selected to be member of theinfected set I . Every node in the graph is considered as starting node. 
2. For each starting node, the following is repeated 50 times:
  1. The simulated time t is incremented by 1. (2.2) For every node in the infected set I , the corresponding outgoing edges are selected as the set E_inf_ .
  2. Each edge in E_inf_ is given a ﬁxed probability (1% in our simulations) that it will infect its sucessor node. If this is true, the corresponding successor node is added to the infected set I.
  3. If the set I contains more than half of the total nodes, t = T_1/2_ is returned. Otherwise, the simulation returns to step 2.
  4. T_1/2_ is stored for averaging and the simulation restarts in step 1 while there are nodes not yet considered as starting points.
3. After all nodes have been considered as starting points, the next set of edges (in 1% blocks, or the following hub for the hub removal strategy) are removed from the graph. The simulation then returns to step 1, until 25% of the edges have been removed.

### 3.5 Testing rewired networks 

Four different rewired versions of the original network were considered: a fully randomized version where only the number of nodes and edges was maintained, a second version which kept the original community structure but randomized the connections inside each community, a third version rewired using the commonly used algorithm which preserves the original degree distribution [23] and a fourth version that kept both the original community structure and degree distribution inside each community.Community structure was obtained using the fast modularity clustering algorithm [24] which identiﬁed four distinct clusters: one for North and Central America, including Canada and Hawaii, another for South America, a third including the greater part of China (except Hong Kong, Macau and Beijing) and ﬁnally a fourth including all other airports. Twenty rewired networks were generated for each version and the results showed the average results of all twenty networks, following the same spreading algorithm as above, with 50 runs for each node as a starting point at each percentage point of removed edges. Therefore each percentage point on the each of the four rewired plots represents the average of 500 000 results.

## Funding information

 Supported by CARMEN e-Science project funded by EPSRC (EP/E002331/1), the Royal Society (RG/2006/R2), and EPSRC PhD studentship (CASE/CNA/06/25) with a contribution from e-Therapeutics plc.

## Competing interests

The authors have declared that no competing interests exist.
